# Secondary data mining of GEO database for long non-coding RNA and Competing endogenous RNA network in keloid-prone individuals

**DOI:** 10.18632/aging.104054

**Published:** 2020-11-16

**Authors:** Yu Deng, Yangbin Xu, Shuqia Xu, Yujing Zhang, Bing Han, Zheng Liu, Xiangxia Liu, Zhaowei Zhu

**Affiliations:** 1 Department of Plastic Surgery, The First Affiliated Hospital of Sun Yat-Sen University, Guangzhou 510080, China

**Keywords:** keloid-prone, lncRNA, GEO database

## Abstract

This study aimed to identify long non-coding RNAs (lncRNAs), microRNAs (miRNAs), and messenger RNAs (mRNAs) differentially expressed (DE) during keloid formation, predict DElncRNA-DEmiRNA-DEmRNA interactions, and construct a competing endogenous RNA (ceRNA) network through secondary data mining of keloid-related sequencing and microarray data in the open-source Gene Expression Omnibus (GEO) database. The GSE113621 dataset was downloaded from the GEO database, |log_2_FC|>1 and p<0.05 were set as screening criteria, genes expressed only in keloid-prone individuals were selected as research objects, and DEmRNAs, DElncRNAs, and DEmiRNAs before injury and 6 weeks after injury were screened. A Pearson correlation coefficient (PCC) of > 0.95 was selected as the index to predict the targeting relationships among lncRNAs, miRNAs, and mRNAs; and a network diagram was constructed using Cytoscape. The expression of 2356 lncRNAs was changed in the keloid-prone group—1306 were upregulated and 1050 were downregulated. Six lncRNAs, namely, 2 upregulated (DLEU2 and AP000317.2) and 4 downregulated (ADIRF-AS1, AC006333.2, AL137127.1 and LINC01725) lncRNAs, were expressed only in the keloid-prone group and were used to construct a ceRNA network. DLEU2 may regulate fibroblast proliferation, differentiation, and apoptosis through hsa-miR-30a-5p/hsa-miR-30b-5p. In-depth mining of GEO data indicated that lncRNAs and a ceRNA regulatory network participate in the wound healing process in keloid-prone individuals, possibly providing novel intervention targets and treatment options for keloid scars.

## INTRODUCTION

Both hypertrophic scars and keloids are pathological scars. Due to abnormal proliferation of dermal fibrous tissue, excessive deposition of collagen during the wound healing process often affects the appearance of patients and even affects local function [1]. Although many studies have addressed the mechanism underlying keloid occurrence and development and the treatment of keloids, the therapeutic effect of existing treatments on keloids is still not ideal [2]. Gene chips and sequencing are powerful tools currently used for studying gene expression profiles; however, due to limitations of the manufacturing process and algorithms for analysis of gene chips during the early stage of this technology, the required data could not be acquired completely [3]. As bioinformatic analysis methods have continually developed and the understanding of epigenetics has increased, many scholars have mined new transcriptome information from old microarray or sequencing data by improving the original algorithms and upgrading the databases to offer new ideas for clinical and scientific research [3–7].

In this study, sequencing and microarray data related to keloid formation were downloaded from the open-source National Center for Biotechnology Information (NCBI) Gene Expression Omnibus (GEO) database, and secondary data analysis was conducted by combining the R language with network database bioinformatic analysis technology. This analysis aimed to identify the coding RNAs (messenger RNAs, mRNAs) and non-coding RNAs (ncRNAs), including microRNAs (miRNAs) and long non-coding RNAs (lncRNAs), expressed during keloid formation and to predict their expression and relationships. Through this research, we intended to verify the possibility of secondary bioinformatic analysis of sequencing and microarray data to extract lncRNAs and construct a competing endogenous RNA (ceRNA) network and simultaneously mine these data for information related to regulation of the pathophysiological process of keloid formation. This study offers new research methods for more comprehensive exploration of keloid pathogenesis and treatment.

## RESULTS

### Changes in mRNAs and ncRNAs in skin wounds of keloid-prone individuals and control individuals 6 weeks after injury

**Figure 1:**
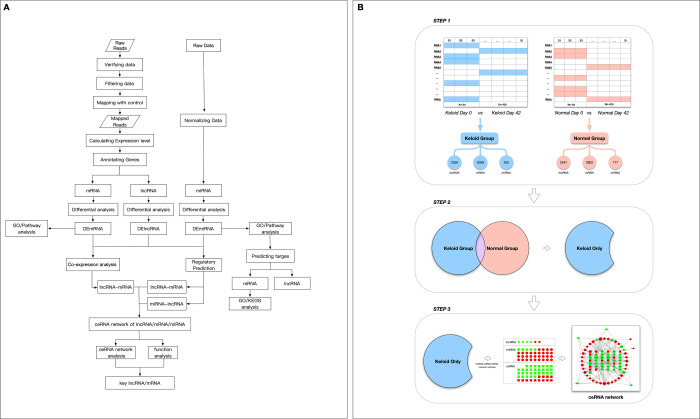
**Volcano plots of mRNA expression in the two groups; GO and KEGG enrichment analyses of specific genes expressed in keloid-prone individuals.** (**A**) mRNA expression in normal individuals. (**B**) mRNA expression in keloid-prone individuals. (**C**) GO enrichment analysis. (**D**) KEGG enrichment analysis. In A and B, the left side of the dashed line represents the uninjured state (day 0); the right side represents the injured state (day 42).

Secondary mining of the RNA sequencing data and miRNA array data was conducted for different individuals. In the 8 keloid-prone individuals, 3039 DEmRNAs were identified—1479 were upregulated and 1560 were downregulated 6 weeks after injury compared with before injury (Figure 1B). Heatmap of lncRNA expression before and after injury in Keloid-prone groups were shown in Figure 1C. In the 6 control group individuals, 2802 DEmRNAs, namely, 1436 upregulated (Figure 1A) and 1366 downregulated mRNAs. A total of 2356 DElncRNAs, namely, 1306 upregulated and 1050 downregulated lncRNAs were identified in the keloid-prone group; 2547 DElncRNAs—1479 upregulated and 1068 downregulated lncRNAs were identified in the control group. Analysis of the miRNA array data revealed 252 DEmiRNAs (151 upregulated and 101 downregulated miRNAs) in keloid-prone patients and 177 DEmiRNAs in the control group (95 upregulated and 82 downregulated miRNAs).

### GO enrichment analysis of mRNAs expressed only in keloid-prone individuals 6 weeks after injury

To more comprehensively study the effect of DEmRNAs on biological activities, BP category analysis was performed. The DEmRNAs were enriched mainly in the BP terms muscle contraction, presynapse assembly, regulation of membrane potential, and presynapse organization. The top 10 enriched terms are listed in Table 1-BP. In the CC category analysis, the DEmRNAs were enriched mainly in the CC terms synaptic membrane, postsynaptic membrane, intrinsic component of synaptic membrane, and glutamatergic synapse. The top 10 enriched terms are listed in Table 1-CC. In the MF category analysis, the DEmRNAs were enriched mainly in the MF terms channel activity, substrate-specific channel activity, passive transmembrane transporter activity, and ligand-gated ion channel activity. The top 10 enriched terms are listed in Table 1-MF. The GO enrichment analysis results are summarized in Figure 1C.

**Figure 2:**
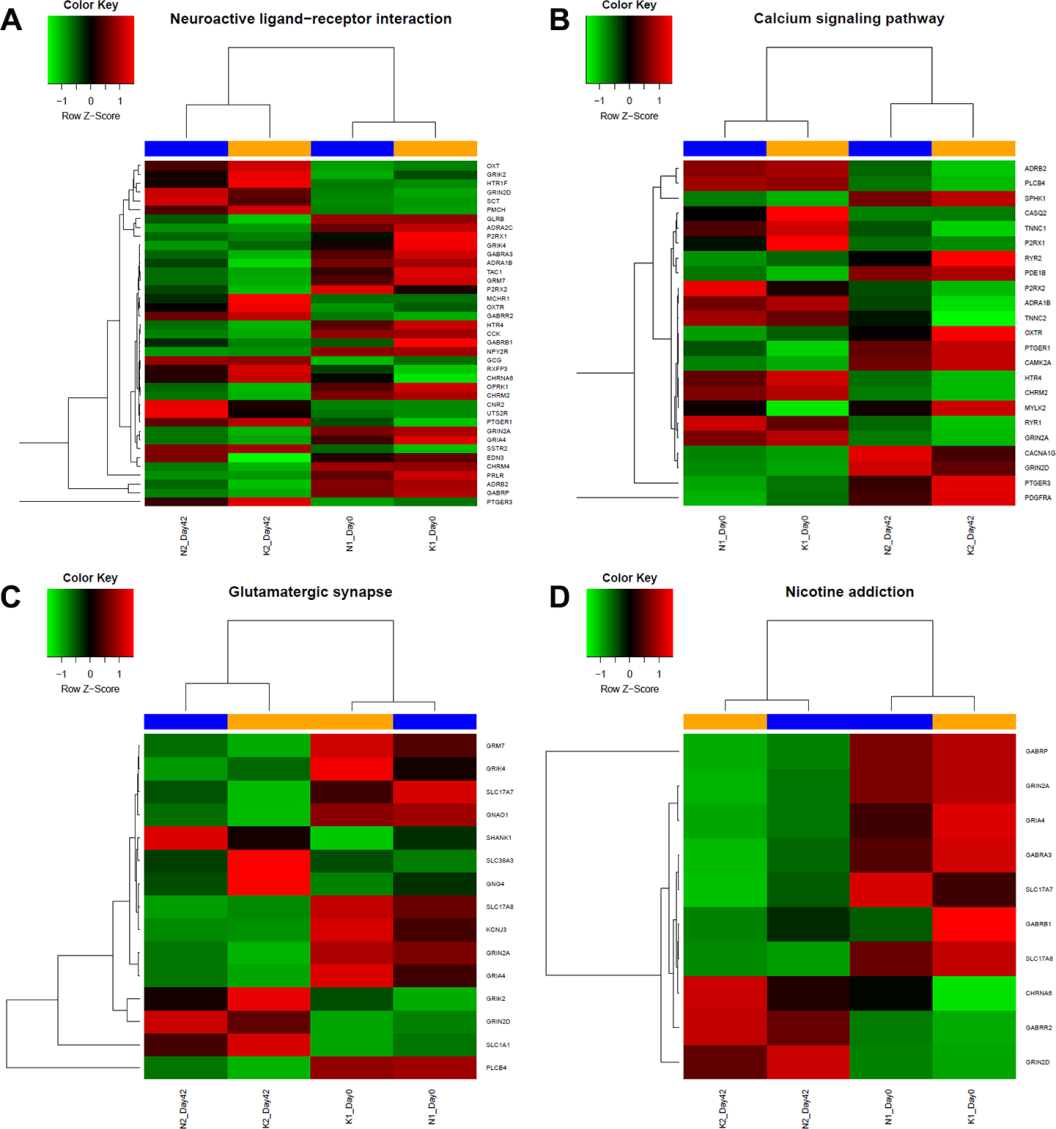
**Heatmap of specific gene expression in different groups form 4 different KEGG enrichment pathway categories.** According to results from KEGG analysis, expression level of genes of each group from (**A**) Neuroactive ligand-receptor related pathways, (**B**) Calcium signalling related pathway, (**C**) Glutamatergic synapse related pathway and (**D**) Nicotine addiction related pathway were shown. Red indicates upregulation; green indicates downregulation; blue indicates the normal group, including day 0 and day 42; yellow indicates keloid-prone individuals, including day 0 and day 42.

**Table 1:** GO Analysis of mRNAs specifically expressed in keloid-prone individuals 6 weeks after injury.

	Term ID	Term description	Ct.	Adjusted p-value
BP	GO:0006936	muscle contraction	35	0.001473
	GO:0099054	presynapse assembly	11	0.001702
	GO:0042391	regulation of membrane potential	37	0.001702
	GO:0099172	presynapse organization	11	0.001702
	GO:0035270	endocrine system development	18	0.001702
	GO:0035418	protein localization to synapse	12	0.002476
	GO:0051952	regulation of amine transport	13	0.002476
	GO:0071772	response to BMP	19	0.003641
	GO:0071773	cellular response to BMP stimulus	19	0.003641
	GO:0015837	amine transport	13	0.003706
CC	GO:0097060	synaptic membrane	45	2.00E-06
	GO:0045211	postsynaptic membrane	37	4.25E-06
	GO:0099240	intrinsic component of synaptic membrane	21	0.001069
	GO:0098978	glutamatergic synapse	34	0.001069
	GO:0005858	axonemal dynein complex	6	0.00128
	GO:0098936	intrinsic component of postsynaptic membrane	17	0.001407
	GO:1902495	transmembrane transporter complex	28	0.001456
	GO:0098793	presynapse	38	0.001534
	GO:1990351	transporter complex	28	0.001534
	GO:0034703	cation channel complex	21	0.001567
MF	GO:0015267	channel activity	37	0.000261
	GO:0022838	substrate-specific channel activity	35	0.000261
	GO:0022803	passive transmembrane transporter activity	37	0.000261
	GO:0015276	ligand-gated ion channel activity	17	0.000513
	GO:0022834	ligand-gated channel activity	17	0.000513
	GO:0005216	ion channel activity	33	0.000517
	GO:0008509	anion transmembrane transporter activity	27	0.000614
	GO:0005261	cation channel activity	27	0.000708
	GO:0015077	monovalent inorganic cation transmembrane transporter activity	27	0.000879
	GO:0046873	metal ion transmembrane transporter activity	32	0.001416

Ct.: number of genes

### KEGG enrichment analysis of mRNAs expressed only in keloid-prone individuals 6 weeks after injury

To more comprehensively analyse the roles played by the DEmRNAs, we performed KEGG pathway analysis. The DEmRNAs were enriched mainly in the neuroactive ligand-receptor interaction, nicotine addiction, calcium signalling pathway, and glutamatergic synapse pathways. The enrichment of DEmRNAs is shown in Figure 1D and Table 2. The gene names and expression heatmaps for each pathway are shown in Figure 2.

**Figure 3:**
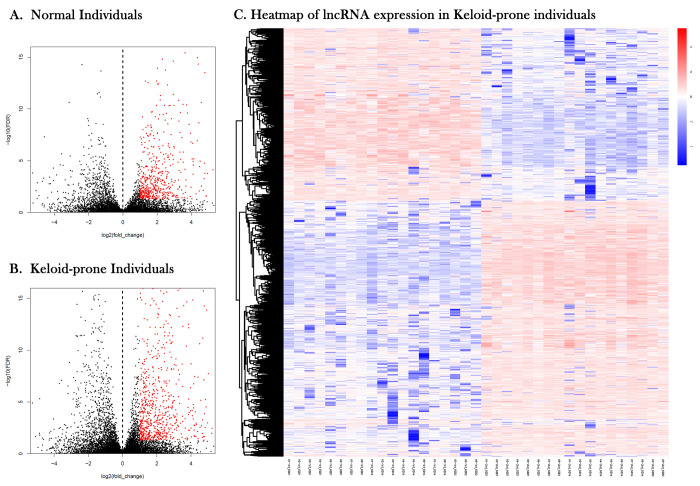
**Volcano plots and heatmap of lncRNA expression in keloid-prone individuals.** (**A**) lncRNA expression in normal individuals. (**B**) lncRNA expression in keloid-prone individual group. In **A** and **B**, the left side of the dashed line represents the uninjured state (day 0); the right side represents the injured state (day 42). (**C**) Heatmap of lncRNA expression before and after injury in Keloid-prone groups.

**Table 2:** KEGG analysis of mRNAs specifically expressed in keloid-prone individuals 6 weeks after injury.

mRNA	Term description	Ct.	Adjusted p-value
hsa04080	neuroactive ligand-receptor interaction	40	3.42×10-6
hsa05033	nicotine addiction	10	0.000953
hsa04020	calcium signaling pathway	23	0.001322
hsa04724	glutamatergic synapse	15	0.010286

Ct.: number of genes.

### Analysis of the lncRNA-miRNA-mRNA network present only in keloid-prone individuals after injury

**Figure 4:**
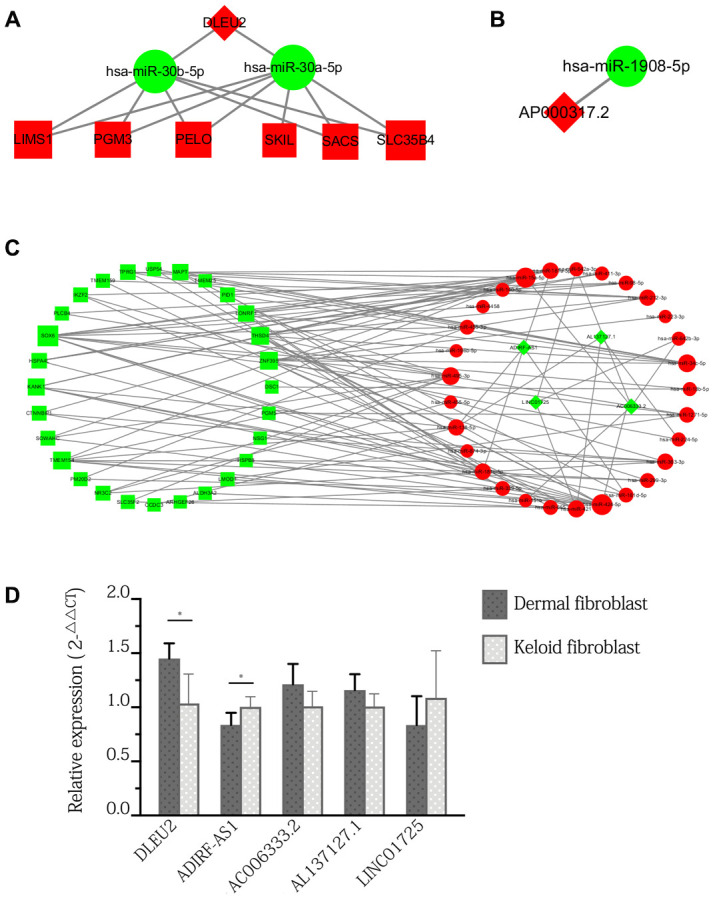
**ceRNA sub-network of specific lncRNAs expressed only in keloid-prone individuals and qPCR verification of DElncRNAs in keloid and normal fibroblasts.** (**A**) Sub-network of DLEU2. (**B**) Sub-network of AP000317.2. (**C**) Sub-network of downregulated lncRNAs. Green indicates downregulated molecules, red indicates upregulated molecules, diamonds indicate lncRNAs, circles indicate miRNAs, and squares indicate mRNAs. (**D**) qPCR verification of DElncRNAs in keloid and normal fibroblasts. *p<0.05.

Volcano plots and heatmap of lncRNA expression in keloid-prone individuals were shown in Figure 3. The StarBase database was used to predict and compare the targets of DElncRNAs and DEmiRNAs, with PCC> 0.95 as the criterion, to construct a lncRNA-miRNA-mRNA molecular interaction diagram (Figure 4A–4C). Six DElncRNAs—2 upregulated (DLEU2 and AP000317.2) and 4 downregulated (ADIRF-AS1, AC006333.2, AL137127.1, and LINC01725)—were screened (Table 3). Additionally, 65 DEmiRNAs were altered (31 were upregulated and 5 were downregulated) as the lncRNAs were altered, and 43 DEmRNAs were altered (13 were upregulated and 30 were downregulated) as the lncRNAs were altered.

**Table 3:** Differentially expressed lncRNAs in keloid-prone individuals 6 weeks after injury. *p*<0.05 indicates a statistically significant difference.

Gene Id	Gene_Name	KDay0	KDay42	log2FC	Adjusted-P	NDay0	NDay42	log2FC	Adjusted-P
ENSG00000231607	DLEU2	306.79	641.38	1.06	1.33×10-17	74.88	66.26	-0.18	1.00
ENSG00000272657	AP000317.2	75.61	183.43	1.28	3.53×10-17	66.42	119.35	0.85	7.08×10-3
ENSG00000272734	ADIRF-AS1	1350.45	552.62	-1.29	2.46×10-29	1251.46	646.56	-0.95	1.24×10-5
ENSG00000272686	AC006333.2	82.88	39.50	-1.07	8.29×10-9	70.48	41.97	-0.75	1.46×10-2
ENSG00000272084	AL137127.1	661.42	240.08	-1.46	2.66×10-18	407.22	223.42	-0.87	7.60×10-5
ENSG00000233008	LINC01725	257.15	121.20	-1.09	1.87×10-13	211.00	134.79	-0.65	4.60×10-2

### Expression of DElncRNAs in fibroblasts in keloid scars and normal skin

Expression of all 6 DElncRNAs except for AP000317.2 was detected in fibroblasts in both keloid scars and normal skin. Specifically, the expression of DLEU2 and ADIRF-AS1 were in significantly different expression levels between fibroblasts and keloid. In contrast, the differences in the expression levels of AC006333.2, AL137127.1, and LINC01725 between the two groups were not statistically significant (Figure 4D).

## DISCUSSION

Keloid scarring is a clinical condition that is difficult to cure. Although keloids are benign tumours, they often extend past the extent of the damage, invading the surrounding normal skin, and seriously affecting the appearance of the skin after healing [4]. Keloids are most common in people between the ages of 10 and 30 years, are especially common in individuals of African, Hispanic, and Asian ethnicity, and frequently occur on the chest, earlobes, shoulders, and back [26]. Although different treatment methods, such as radiotherapy, hormone therapy, and surgical resection are available, keloids still have a relatively high recurrence rate [8]. Many scholars believe that fibroblasts are a main participant in the occurrence and development of keloids. After skin injury, a complicated regulatory signalling network is activated to regulate the proliferation, migration, and secretion of fibroblasts. Therefore, the biological behaviour of fibroblasts regarding the mechanism of keloid formation is a trending research topic [9]. Gene chips and sequencing are important methods for studying gene transcription profiles and are widely used in regenerative medicine [30], disease [27, 10–11], and cancer [7] research. However, the focus of transcriptome research has recently shifted gradually from protein-coding genes to epigenetics involving ncRNAs. In 2018, Onoufriadis et al. [26] compared patients with keloids with patients without keloids. They sampled wounds on the buttocks of all participants and sampled the untreated wounds 6 weeks later. Two specimens from each participant were obtained for transcriptome analysis, and the results revealed differences in mRNA and miRNA expression between the 2 groups and identified the miRNA-mRNA regulatory relationships. In the current study, the open-source GEO database to conduct secondary mining of ncRNAs and mRNAs through bioinformatics algorithms, aiming to identify suitable ncRNA molecules for further exploration of the epigenetic regulation mechanism of keloids.

In this study, the genes of keloid-prone patients were separated from those of control individuals and were analysed separately according to the wound healing process. Considering that the wound healing process is common to all populations, we excluded the intersecting set of genes between the keloid-prone patients and the control individuals, retaining only the genes differentially expressed in keloid-prone patients for further study. Because differential gene screening is generally based on counts, errors often occur. To reduce the false positive and false negative rates, the most recent version of DESeq2 software was used herein to obtain the required DEmRNAs and DElncRNAs in order to ensure the accuracy of the data analysis [12]. For typical prediction of miRNA target genes, more than 2 miRNA target gene prediction software programs are selected for prediction, and the intersection of the prediction results is then obtained to identify the most reliable miRNA target gene prediction results [26]. In this study, we used the StarBase database for analysis. The miRNA data in this database were verified by Ago2-CLIP-Seq [31] with a relatively high degree of reliability.

GO is an international standardized gene functional classification system that provides a set of dynamically updated controlled terms to comprehensively describe the attributes of genes and gene products in organisms. GO has 3 ontologies, which individually describe the MF, CC, and BP of genes. The basic unit of GO is the term, and each term corresponds to an attribute. Each gene is associated with 1 or more GO terms (aka GO functions) [13]. KEGG pathway analysis is a gene annotation database-based method for detecting pathways significantly enriched with differentially expressed genes. Therefore, a comprehensive database and complete pathway annotations are the keys to pathway analysis. Pathway analysis results reveal direct interactions between genes in the actual signalling pathways. Compared with GO analysis, pathway analysis allows researchers to study the biological effects of differentially expressed genes; moreover, it is complementary to GO analysis [30].

This study showed that during wound healing, DEmRNAs expressed only in keloid-prone individuals were mainly enriched in various ion channels in GO MF categories. KEGG pathway analysis further showed that these DEmRNAs were enriched mainly in the calcium signalling pathway. Liang et al. reported a similar finding in a microarray study focusing on DElncRNAs and DEmRNAs between keloid tissue and normal skin tissue and found that the lncRNA CACNA1G-AS, an antisense RNA to CACNA1G, which encodes a calcium channel subtype, was upregulated in keloid tissue in independent pairs of samples [14]. Calcium channels are associated with reactions to mechanical forces in different cell types, and elevated intracellular calcium levels were observed in fibroblasts and keratinocytes under application of hydraulic pressure [15]. Promotion of calcium channel expression could positively affect the migration of keloid fibroblast cells [16]. L-type calcium channel blockers, such as verapamil, have already been used in clinical treatment for keloids and have been proven to be safe and effective [17]. These results collectively suggest that dysregulation of a mechanical signal-related network, with mediation of calcium channel activation, could be pivotal in keloid development. KEGG pathway analysis also showed that the glutamatergic synapse pathway might also play an important role. Evidence has shown immunoreactivity of L-glutamate and its ionotropic receptor NMDAR in the human epidermis [18–20]. These effects may cooperatively regulate the calcium levels in related tissues and cells, as previously reported [21]. Moreover, our findings showed that neuroactive ligand-receptor interactions might also contribute. A 2018 bioinformatic analysis of the molecular mechanism of pathological scarring by Zhang et al. noted that genes expressed in scar tissues were enriched in this channel [22]. The neuroactive ligand-receptor interaction pathway includes hundreds of genes involving many types of neuroreceptors, suggesting a broader, yet-to-be-discovered, complex neuroactive ligand-receptor mediated network; therefore, further investigations are needed. Moreover, GO analysis showed that the genes were enriched not only in the BP terms muscle contraction, presynapse assembly, and regulation of membrane potential but also CC terms related to different structural parts of synapses. Last, by KEGG analysis, we also found DEmRNAs enriched in the nicotine addiction pathway, which has not been reported in previous studies and awaits experimental verification.

ncRNAs are RNAs that are not directly translated into proteins and were once thought to be non-functional components in gene expression and transcription [10]. As epigenetics and other research methods for genes and proteomic approaches have recently been developed, ncRNAs have been found to be associated with gene expression, not only regulating gene transcription and post-transcriptional modifications and translation but also forming ceRNA regulatory networks to participate in mutual reciprocal regulation, which in turn affects the biological functions of cells, tissues, and organisms [20]. According to their length, ncRNAs can be divided into small non-coding RNAs (sncRNAs), lncRNAs, and circular RNAs (circRNAs). Wang et al. studied the expression and effects of lncRNA-H19 in keloid fibroblasts and found that H19 regulated the viability and apoptosis of fibroblasts through the miR-29a/COL1A1 axis [19]. However, the functions of most lncRNAs are still unclear. Therefore, we used the existing gene data for keloid fibroblasts to identify the ncRNA molecules differentially expressed during keloid formation and to more comprehensively explore the epigenetic regulatory mechanism in keloids.

We found that a total of 6 lncRNAs were involved in the network: ADIRF-AS1, DLEU2, AP000317.2, AC006333.2, AL137127.1, and LINC01725. Among these DElncRNAs, DLEU2 and AP000317.2 were upregulated, and the others were downregulated. The ceRNA sub-network in which these lncRNAs are involved is shown in Figure 4A–4C. The experimental design for acquisition of the original GEO data involved invasive surgery on uninjured skin of patients with keloid scars. Therefore, no matching data can be found in public databases. To verify the expression of the DElncRNAs, we used pathological specimens of keloid scars as a substitute to compare with normal skin. The results showed that only the expression of DLEU2 and ADIRF-AS1 in keloid fibroblasts showed significant difference and that the expression of other genes was not changed significantly. We cannot exclude the possibility that our results were affected by differences in the sequencing models or qPCR analysis. Although these lncRNAs have not been reported in previous keloid-related literature, molecules such as DLEU2 and ADIRF-AS1 have been detected in other tissues and diseases [23, 24]. DLEU2, a gene located on chromosome 13q14, has frequently been observed to be deleted or epigenetically suppressed in leukaemia and is considered a tumour suppressor [24, 25]. Wu et al. found that the pattern of high lncRNA DLEU2 expression or low miR-30a-5p expression was an unfavourable prognostic factor for survival and tumour recurrence in patients with non-small cell lung cancer and showed that the lncRNA DLEU2 accelerates the development of cancer by sponging miR-30a-5p [24]. In this study, we predicted that DLEU2 acted on target genes, including development-related genes (such as LIMS3), apoptosis-related genes (such as SKIL), differentiation-related genes (such as PELO), and protein transporter-related genes (such as SLC35B4), through miR-30a-5p or miR-30b-5p, suggesting that DLEU2-associated ceRNAs may play an important regulatory role in keloid formation and wound healing. This possibility awaits verification by more basic and clinical experimental data.

Through data mining of the open-source GEO database, we acquired new regulatory information from previous sequencing and microarray data. However, our study has limitations. First, our study was based on secondary analysis of online sequencing data with limited amounts of original clinical samples. More clinical samples are needed to provide supportive evidence. The differential expression of lncRNAs and their targets in our ceRNA network also requires confirmation via qRT-PCR. Second, our hypotheses were inspired by similar findings in tumour studies, because keloids are thought—but have not been confirmed—to be tumour-like. Therefore, further well-designed experiments are needed to verify these hypotheses.

## CONCLUSION

This study used bioinformatics methods to perform in-depth data mining of the ncRNA data in GEO microarray datasets. We found that the calcium signalling pathway, glutamatergic synapse pathway, neuroactive ligand-receptor interaction pathway and nicotine addiction pathway may have potential regulatory effects that influence keloid development. We also identified lncRNAs and a ceRNA regulatory network that exist only in keloid-prone patients during the wound healing process, possibly providing novel intervention targets and therapeutic options for patients with keloid scars.

## MATERIALS AND METHODS

### Acquisition of GEO sequencing data

The GSE113621 dataset, including sequencing data from the GSE113619 dataset and raw miRNA microarray data from the GSE113620 dataset, was acquired from the NCBI GEO database (http://www.ncbi.nlm.nih.gov/geo). This dataset included data from 8 keloid-prone patients (K1-K8) and 6 non-keloid-prone patients (N1-N6). The specific information for the patients is listed in Table 4 [26]. The data can be divided into 4 groups, where K_1-8_-1^st^ refer to tissues from keloid-prone patients before injury, K_1-8_-2^nd^ refer to tissues from keloid-prone patients 6 weeks after injury, N_1-6_-1^st^ refer to tissues from non-keloid-prone patients before injury, and N_1-6_-2^nd^ refer to tissues from non-keloid-prone patients 6 weeks after injury. The data were acquired on the Illumina HiSeq 2500 (*Homo sapiens*) and Illumina HiSeq 3000 *(Homo sapiens*) sequencing platforms, and the Affymetrix Multispecies miRNA-4 Array microarray data platform was used. The analysis process is shown in Figure 5A and 5B.

**Figure 5:**
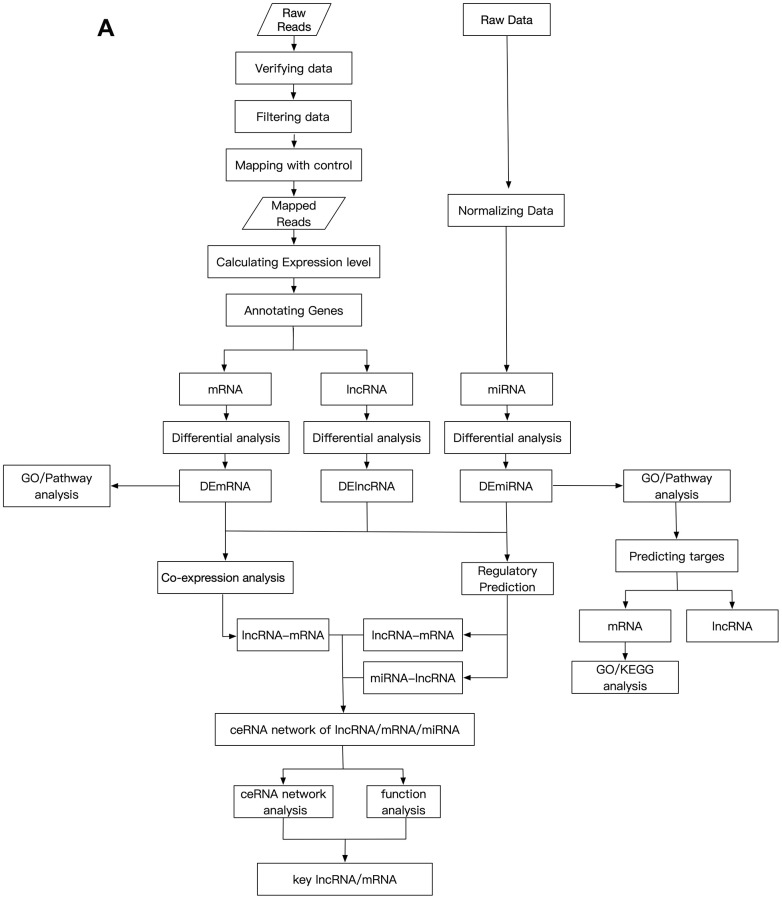
**Schematic design of the study.** (**A**) Data processing and analysis procedures.

**Figure 5:**
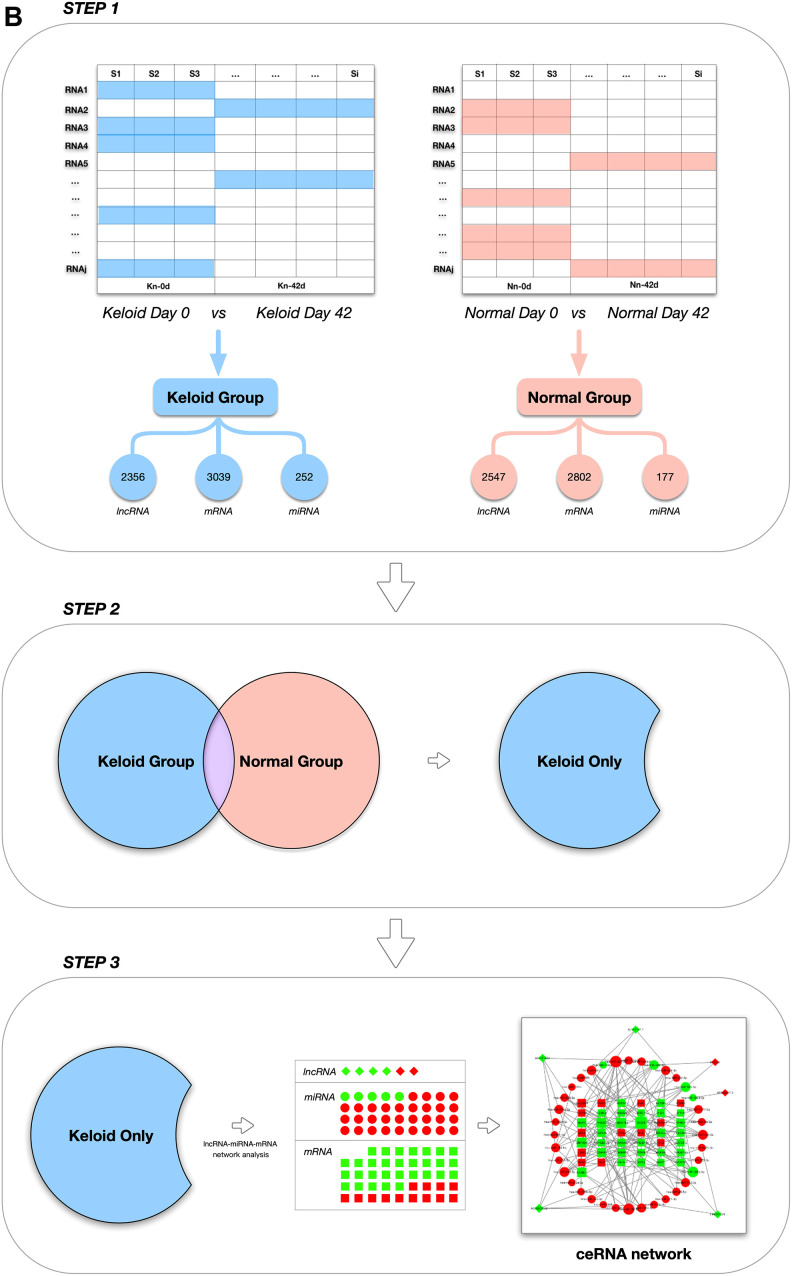
**Schematic design of the study.** (**B**) Grouping diagram.

**Table 4:** Details of patients in GEO dataset SRP137071.

Keloid-prone ID	Age (years)	Sex	Control ID	Age (years)	Sex
K-1	32	male	N-1	24	male
K-2	23	male	N-2	58	male
K-3	28	female	N-3	28	male
K-4	23	male	N-4	22	male
K-5	24	male	N-5	31	male
K-6	30	male	N-6	34	male
K-7	57	female			
K-8	25	male			

### Quality control and annotation of sequencing data

The raw RNA sequencing (RNA-Seq) data in FASTQ format were acquired from the GEO database and initially filtered using FastQC (https://www.bioinformatics.babraham.ac.uk/projects/fastqc/) to obtain the sequencing quality distribution, base content distribution, and proportion of repetitive sequencing fragments [27]. Because this portion of the data was basically normal, thus having no direct impact on the results, no QC results were provided. After quality control, Hisat2 software was used to compare the clean reads with the reference genome (hg38), and the count rate of each sample was compared [28]. According to the comparison results, the fragments per kilobase per million mapped reads (FPKM) value was used as the standard for quantitation of gene expression, and mRNAs and lncRNAs were quantitatively analysed based on the annotation information in the Genecode V25 database.

### Screening and statistical analysis of mRNAs differentially expressed between different groups

To more comprehensively study phenotype-related gene expression, the differentially expressed genes between an experimental group and the control group must be identified and correlated with a phenotype. For differential gene expression analysis, the most recent version of DESeq2 software was used, and the thresholds for significant differential gene expression were set as follows: |log_2_FoldChange (FC)|>1 and FDR<0.05 [29]. As shown in Figure 5B, differentially expressed mRNAs and lncRNAs (DEmRNAs and DElncRNAs) in the K_1-8_-1^st^/K_1-8_-2^nd^ and N_1-6_-1^st^/N_1-6_-2^nd^ groups were screened first and designated as the keloid group and the non-keloid-prone (control) group, respectively. DEmRNAs and DElncRNAs that were also expressed in the control group were excluded from the keloid group, and genes that were expressed only in the keloid group were selected for subsequent analysis.

### Gene Ontology (GO)/Kyoto Encyclopedia of Genes and Genomes (KEGG) functional enrichment analyses of DEmRNAs

The analysis method [30] previously described by our group was used to perform GO and KEGG pathway enrichment analysis. In brief, DEmRNAs were used as the analysis objects, and to obtain all GO terms corresponding to each DEmRNA, GO annotation analysis based on the GO database was performed for 3 ontologies: biological process (BP), molecular function (MF), and cellular component (CC). Fisher’s exact test was used to calculate the significance level of each GO term. The test criterion α was set to 0.05, and the significantly enriched GO terms were screened.

Additionally, KEGG pathway annotation was performed for the DEmRNAs in the database to obtain all pathway terms corresponding to each gene. The significance level of each pathway term was calculated using Fisher’s exact test. The test criterion α was set to 0.05, and significantly enriched pathway terms were screened. Heatmaps of pathway terms highly enriched with the DEmRNAs were generated using the R language.

### Secondary mining of miRNA microarray data

The raw data for miRNA expression analysis were obtained from a GEO dataset (GEO accession number GSE113621). In brief, the CEL file was pre-processed and normalized with the R packages affy (version 1.64.0) and simpleaffy (version 2.62.0). Differential expression analysis of miRNAs was performed with the R package limma (version 3.42.2). MiRNAs for which |FC| >1.5 and P<0.5 were considered significant DEmiRNAs. MiRNA target prediction was performed with StarBase version 3.0 (http://starbase.sysu.edu.cn) [31, 32].

### Data acquisition and construction of the lncRNA-miRNA-mRNA ceRNA network

After the DElncRNA, DEmiRNA, and DEmRNA analysis results and miRNA target gene prediction results were obtained, the lncRNA-miRNA-mRNA ceRNA network diagram was constructed [33, 34] by the following method. First, the Pearson correlation coefficient (PCC) was used to evaluate the correlations between the expression of DElncRNAs and DEmRNAs. PCC> 0.95 was used as the screening criterion, and the screened lncRNA-mRNA combinations were considered correlation pairs. Next, miRNA target genes were screened from the lncRNA-mRNA correlation pairs. For lncRNA-mRNA co-expression pairs that were simultaneously negatively correlated with an miRNA and were also target genes of the miRNA, the lncRNA-mRNA-miRNA axis was considered a competitive triad. Finally, Cytoscape (version 3.6.1) was used to visualize the ceRNA competitive triad in the network.

### qPCR verification of DElncRNA expression in fibroblasts from keloid scars and normal skin

The expression levels of deleted in lymphocytic leukaemia 2 (DLEU2), AP000317.2, ADIRF-AS1, AC006333.2, AL137127.1, and LINC01725 were measured at different time points using real-time qPCR. In brief, keloid tissue and normal skin were collected from different patients (n=5). This study conformed to the guidelines established by the Ethics Committee of the First Affiliated Hospital of Sun Yat-sen University, and written informed consent was obtained from all included patients. Via the extraction method described in Han et al. [2], fibroblasts were extracted from both groups of tissues and were subcultured in an incubator. Total RNA was extracted using TRIzol (Thermo Fisher Scientific, USA). The RNA concentration and purity were measured using a NanoDrop 2000 (Thermo Fisher Scientific), and the final concentration was adjusted to 200 ng/μl. RNA (1 μg from each specimen) was reverse transcribed with a RevertAid First Strand cDNA Synthesis Kit (Thermo Fisher Scientific). An appropriate amount of cDNA was amplified using FastStart Universal SYBR Green Master Mix (Roche, Switzerland) in a StepOnePlus Real-Time PCR instrument (Thermo Fisher Scientific). The specific procedure is shown in the system manual. Three replicates per specimen were analysed. The expression levels of all target mRNAs were normalized to those of Gapdh. The information about the detected genes and the amplification primers are shown in Table 5. The specificity of the RT-PCR results was confirmed via routine agarose gel electrophoresis and melting curve analysis. The 2-ΔΔCt method was used to calculate relative gene expression levels.

**Table 5:** qPCR primer sequences of DElncRNAs specifically expressed in keloid-prone individuals.

LncRNA	Forward (5’-3’)	Reverse (5’-3’)
DLEU2	TCC GAG AGT ATA GCG CCA CT	ACT GCC CTT TGC TCC AAG TA
ADIRF-AS1	GCC CAC TGA ATT CCC CTG AA	AAT CAG AGT GAC TGC CCC AC
AC006333.2	TCC AAC TTC GTA CTC TGG CC	GCT TCG CAA AGG TGT ACG TC
LINC01725	TCC AGC TCT TCT CCC CTG AA	GGG GGA ACT TAG AAA GGC CA
AP000317.2	AGT GGA TGG CAA GCT TCC TT	GGA ACC CTC GTC TTT GGG AA
AL137127.1	TCC CTT GAA TGC ACA GCC AT	CAA TGT GGT GCC CCA ACT TG
